# Structural basis of the RNA-mediated Retron-Eco2 oligomerization

**DOI:** 10.1038/s41421-025-00823-y

**Published:** 2025-09-02

**Authors:** Yanjing Wang, Chen Wang, Yongqi Yin, Yongqing Cui, Zhikang Dai, Chang Liu, Yanke Chen, Zeyuan Guan, Tingting Zou

**Affiliations:** 1 National Key Laboratory of Agricultural Microbiology, Hubei Hongshan Laboratory, Huazhong Agricultural University, Wuhan, Hubei China; 2 Department of Pathology, UMass Chan Medical School, Worcester, MA USA; 3 National Key Laboratory of Crop Genetic Improvement, Hubei Hongshan Laboratory, Huazhong Agricultural University, Wuhan, Hubei China

**Keywords:** Cryoelectron microscopy, Long non-coding RNAs

## Abstract

In the evolutionary arms race between bacteria and viruses, retrons have emerged as distinctive antiphage defense systems. Here, we elucidate the structure and function of Retron-Eco2, which comprises a non-coding RNA (ncRNA) that encodes multicopy single-stranded DNA (msDNA, a DNA‒RNA hybrid) and a fusion protein containing a reverse transcriptase (RT) domain and a topoisomerase-primase-like (Toprim) effector domain. The Eco2 msDNA and RT-Toprim fusion protein form a 1:1 stoichiometric nucleoprotein complex that further assembles into a trimer (msDNA:RT-Toprim ratio of 3:3) with a distinctive triangular configuration. The RNA portion of the msDNA in one protomer closely intertwines around the RT domain of an adjacent protomer, mediating the formation of this self-inhibitory assembly. Upon activation, the Toprim effector domain exhibits RNase activity, degrading RNA to arrest phage replication. We further reveal that phage mutants evading Eco2-mediated defense harbor mutations in the endonuclease IV-like protein DenB, underscoring DenB’s critical role in triggering the activation of this system. Together, these findings provide key structural and functional insights into Retron-Eco2, laying the groundwork for harnessing its potential in biotechnology and synthetic biology applications.

## Introduction

In the ongoing co-evolutionary arms race between prokaryotes and their viral parasites, bacteria have evolved a multitude of sophisticated antiviral immune systems with diverse defense mechanisms^1–8^. Among these immune systems, retrons, a class of widespread genetic elements found in ~11% of the bacterial genomes, along with their associated effector proteins, have recently emerged as key players participating in antiphage defense^3,9–13^. A typical retron comprises a reverse transcriptase (RT) and a non-coding RNA (ncRNA)^14^. The retron RT reverse-transcribes the msd segment of the ncRNA (msdRNA) into DNA (msdDNA), which, together with the msr segment (msrRNA), forms a single-stranded branched DNA‒RNA hybrid named multicopy single-stranded DNA (msDNA)^9,15–19^. Beyond their biological roles, retrons have attracted interest as versatile tools for genome engineering due to their ability to synthesize msDNA^20–33^.

Retrons can be classified into multiple types based on their associated effector proteins^9,10^, yet the molecular details of their antiphage mechanisms remain largely obscure. While a few systems have been characterized, such as Retron-Eco1 (also known as Ec86) that forms filamentous complexes and mediates antiphage defense via NAD^+^ hydrolysis^12,34,35^, and Retron-Eco6 (Ec48) that compromises membrane integrity^9^, the underlying defense mechanisms of most retron systems remain poorly understood. This knowledge gap is especially pronounced for retrons where the RT and effector reside within a single fusion protein to function.

Several antiphage/antiplasmid defense system-related proteins contain integral topoisomerase/primase (Toprim) domains, including those found in the overcoming lysogenization defect (OLD) family proteins^36^, Detocs^37^, Gabija^1,38–41^, Wadjet^42–45^, PARIS^46–48^, and retron systems^3,9,10^. Among Type I retrons, Eco2 (also known as Ec67) stands out for its RT-Toprim fusion protein^3,9,49^. Previous studies have indicated that Eco2 assembles into large supramolecular complexes^50^. Investigating the structural basis of Eco2-mediated defense and unraveling its mode of action will provide critical insights into the broader functional roles of Toprim domains in bacterial immunity.

Here, we purify and characterize the Retron-Eco2 system from *Escherichia coli* Cl-1 and determine its cryo-electron microscopy (cryo-EM) structure at 2.6 Å resolution. We find that Eco2 forms a triangular trimer comprising three msDNA-RT-Toprim units. The RNA part of msDNA interconnects RT domains across adjacent protomers, creating a self-inhibitory complex. Toprim domains, arranged as three legs extending from the msDNA-RT core, exhibit RNase activity upon activation, degrading RNA to arrest phage replication. Genetic analyses confirm the importance of Toprim enzymatic activity and implicate the phage-encoded DenB endonuclease IV-like protein in activating Eco2-mediated defense. Although Eco2 activation leads to translational inhibition, the underlying mechanism remains to be explored.

## Results

### Structural determination of the Retron-Eco2 defense system

We first analyzed 3395 retron/retron-like RTs sequences and constructed a comprehensive phylogenetic tree (Supplementary Fig. S1). Notably, ~32.8% of these RT proteins are fused with effector domains, such as Toprim, protease, and Toll/interleukin-1 receptor (TIR) domains. Among these, the Toprim domain is the most prevalent, accounting for 37% of the effector associations, as seen in Retron-Eco2. Based on this observation, we focused our structural and biochemical studies on Eco2 to elucidate its function.

**Fig. 1:**
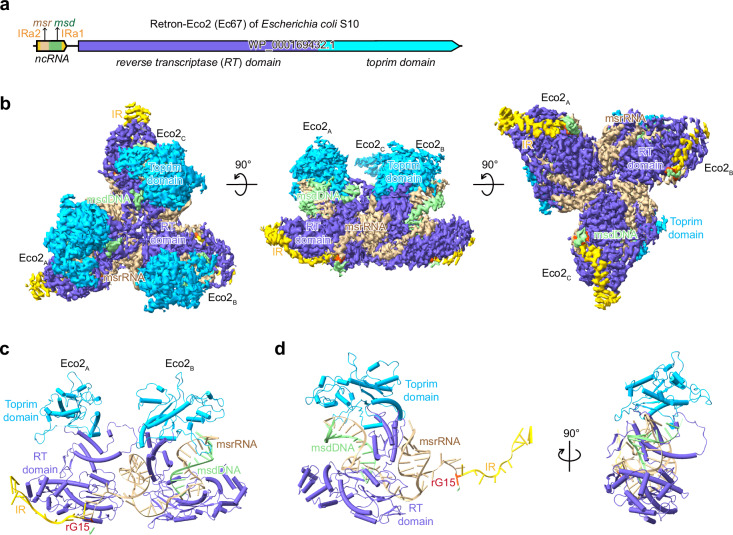
Overall structure of the Retron-Eco2 complex. **a** A schematic diagram of the Retron-Eco2 system derived from *E. coli* strain S10, showing the ncRNA and the RT-Toprim fusion protein used in this study. **b** Cryo-EM density map of the Retron-Eco2 complex, revealing a triangular trimer composed of three RT-Toprim proteins and three msDNA. The msrRNA portion is in tan, msdDNA in light green, IRs in gold, rG15 in orange-red, the RT domain in slate blue, and the Toprim domain in deep sky blue. **c** Close-up view illustrating how each msrRNA bridges two distinct RT-Toprim monomers, spanning from one protomer to the next. The msrRNA occupies the RT active site in a head-to-tail manner. **d** Cylinder (cartoon) representation of one protomer of the Retron-Eco2 complex, consisting of the RT-Toprim fusion protein (slate blue, deep sky blue) and its corresponding msDNA (tan/light green/orange-red/gold).

To elucidate how the Retron-Eco2 system defends against phage infection, we first cloned and purified the RT-Toprim fusion protein from *E. coli* S10 (Fig. 1a). In contrast to the challenging purification of the Eco1 RT protein, we found that the Eco2 RT-Toprim fusion protein could be readily purified, which was eluted at the volume corresponding to the monomeric form during size exclusion chromatography (SEC) (Supplementary Fig. S2a). Subsequently, we co-expressed and purified RT-Toprim together with its ncRNA, and observed that the Retron-Eco2 complex assembled into an oligomeric complex (Supplementary Fig. S2a), consistent with previous reports^50^. The presence of msDNA in the Eco2 complex was confirmed by UV absorbance at a wavelength of 260 nm and urea-polyacrylamide gel electrophoresis (urea-PAGE) analysis.

To further investigate the assembly of the Retron-Eco2 complex, we determined its cryo-EM structure at a resolution of 2.6 Å (Supplementary Fig. S2b‒f and Table S1). Overall, Retron-Eco2 forms a trimeric, cyclic assembly composed of three msDNA molecules and three RT-Toprim proteins (three msDNA-RT-Toprim units). In the Retron-Eco2 trimer, the msDNA-RT portions from each protomer together adopt a triangular architecture, with the Toprim domains protruding as three perpendicular “legs” extending from this triangular base (Fig. 1b). The three RT-Toprim proteins are held together by the msDNA molecules. The msrRNA part of each msDNA molecule spans from one RT-Toprim to the next in a head-to-tail fashion, occupying the active sites of the RT domain (Fig. 1c, d). Collectively, these findings demonstrate the structural basis of the Eco2 complex.

### Eco2 msDNA adopts a three-bladed propeller-like structure

**Fig. 2:**
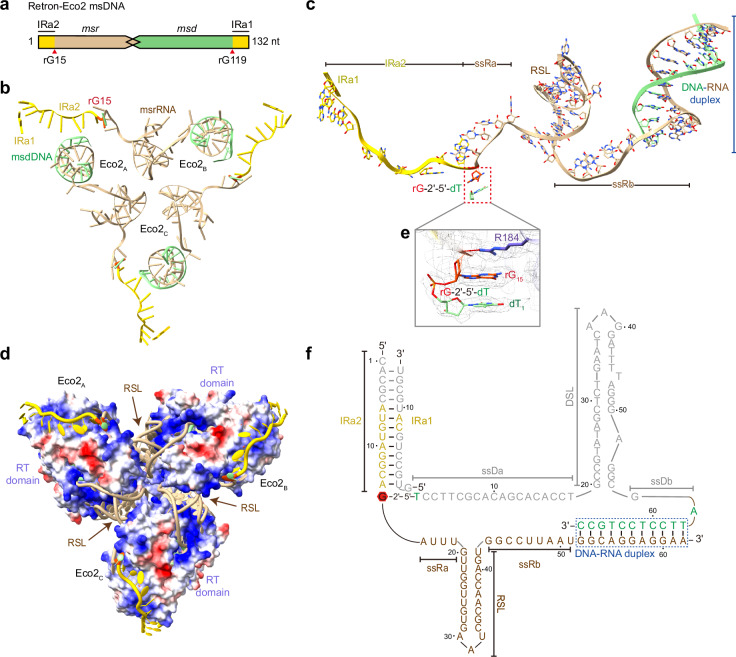
Structure of Retron-Eco2 msDNA. **a** Schematic representation of Eco2 msDNA, showing the complementary IR regions (IRa1, IRa2), the msrRNA region, and the msdDNA region. **b** Structure of the trimeric msDNA with the msdDNA portion in light green, msrRNA in tan, IRs in gold, and rG15 in orange-red, highlighting the propeller-like arrangement. **c** Illustration of a single msDNA protomer, showing most of the msrRNA (IRa1 (rC8–rA9), IRa2 (rA6–rA14), rG15, ssRa (rA16–rU19), the stem-loop (rG20–rU42), ssRb (rG43–rU51), and rG52–rA62 in the RNA‒DNA duplex). dT1, which is linked to rG15 via a 2′,5′-phosphodiester bond, and bases dA56–dC67 of the msdDNA portion are also visible. **d** Electrostatic surface representation of the RT domain, with msDNA shown in cartoon form, highlighting the RNA‒protein interface. **e** Cryo-EM density corresponding to the branched guanosine (rG15) that links to dT1 at the 5′-end of msdDNA via a 2′,5′-phosphodiester bond (orange-red). **f** Secondary-structure diagram showing the color scheme consistent with **b**‒**d**. Unresolved regions are shown in gray, while the blue dotted box encloses the DNA‒RNA duplex.

Although different retrons produce msDNAs with varying primary sequences, they share structural and functional features^10^. Similar to other reported msDNAs, the IRa1/a2 regions at the 5′- and 3′- ends of the Eco2 ncRNA are complementary and can hybridize. The Eco2 msr region, which is not reverse transcribed, forms a single short RNA stem-loop (RSL), while the msd region, composed of 67 bases, folds into a single long DNA hairpin (DNA stem-loop, DSL) (Fig. 2).

Within the Eco2 complex, the msrRNA segments of the three msDNA molecules form a three-bladed propeller-like structure (Fig. 2b). Each msrRNA comprises a 23-bp RSL (rG20‒rU42) flanked by two single-stranded tails, designated the 5’-end tail and the 3’-end tail (Fig. 2c, f). The 5’-end tail includes IRa2, rG15, and the single-stranded RNA a (ssRa) segment (rA16‒rU19), while the 3’-end tail consists of the ssRb (rG43‒rU51) and the RNA portion of the DNA‒RNA duplex. These tails and the RSL region interact tightly with the positively charged surface of the RT domain, stabilizing the trimeric assembly (Fig. 2d). Each msrRNA tail in one protomer associates with an RT-Toprim from another via electropositive grooves, forming hydrogen bonds between the protein and the RNA bases (Fig. 2d).

In the solved structure, a 9-nt IRa2 is clearly visible, while only two complementary bases (rC8 and rA9) of IRa1 are resolved (Fig. 2c, f). The 2′,5′-phosphodiester bond between the branched guanosine (rG15) and dT1 (the 5′-end of the msdDNA) is observed in the density map (Fig. 2c, e, f). The final 11 nucleotides (rG52‒rA62) at the 3′-end of msrRNA and the nucleotides dT57‒dC67 of msdDNA form the DNA‒RNA duplex, which is located within the RT active site (Fig. 2c, f). This configuration shows more paired bases between the msrRNA template and msdDNA product compared to previous reports^49,51^. Except for dT1 and the DNA part of the RNA‒DNA duplex, the cryo-EM density of the remainder of the msdDNA is weaker, indicating less stable binding and increased flexibility. Such flexibility, particularly in the DSL region, may facilitate its role as a sensor of phage infection^9,11,34,35^.

### Retron-Eco2 RT adopts a right-hand-like fold

**Fig. 3:**
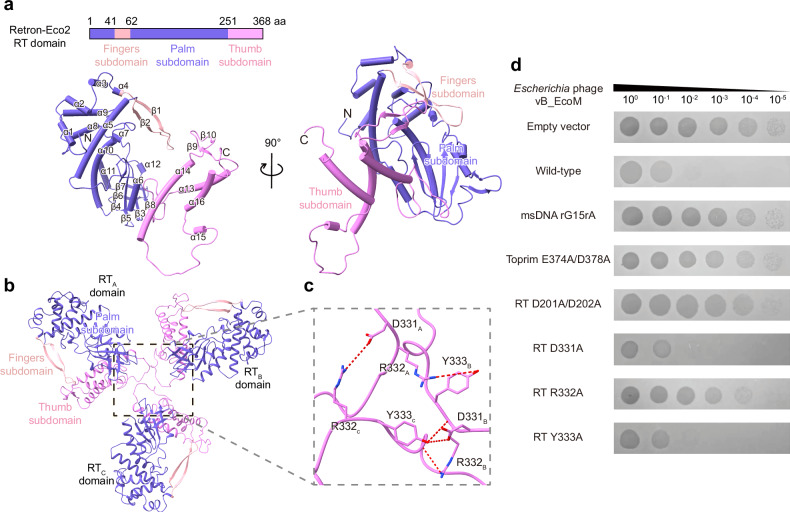
The trimeric structure of the RT domain. **a** The domain organization and atomic model of the Eco2 RT domain with matching colors: palm subdomain (slate blue), fingers subdomain (light pink), and thumb subdomain (violet). The α-helices and β-strands are labeled; “N” and “C” indicate the termini. **b** Three RT domains from the Eco2 complex form a triangular assembly, each interacting with adjacent RT monomers. **c** Close-up view of trimer interface showing key hydrogen-bonding interactions (red dashed lines) among residues D331, R332, and Y333 as sticks. **d** Serial dilution plaque assays of *Escherichia* phage vB_EcoM on *E. coli* MG1655 cells expressing either wild-type or mutant Eco2. The images are representative of three replicates.

The full-length Eco2 RT-Toprim protein consists of an N-terminal RT domain (residues 1‒368) and a C-terminal Toprim domain (residues 369‒586). The N-terminal RT domain adopts the characteristic right-hand-like fold found in all other known RT structures, consisting of 16 α-helices and 10 β-strands (Fig. 3a). Structural alignment indicates that the Eco2 RT domain is related to non-LTR RTs, including those from group II introns, human long interspersed element-1 (LINE-1) elements, insect R2 elements, and other eukaryotic non-LTR retrotransposons^52–55^. DALI analysis further reveals structural similarities to RNA-directed RNA polymerases identified in various RNA viruses (Supplementary Fig. S3a).

We compared several representative RTs from different organisms based on DALI analysis. While the core RT fold is conserved, variations occur in each homologous structure. For example, group IIC introns, DRT2, and LINE-1 possess an additional N-terminal extension (NTE) domain, while Abi-P2 replaces the thumb domain with a helical domain (Supplementary Fig. S3b–i). Among non-retron family RTs, Eco2 RT domain shows the highest structural similarity to the prokaryotic DRT2 defense system, which can reverse-transcribe neighboring noncoding RNA into tandem repeats that facilitate the expression of a toxic repetitive protein^56^ (Supplementary Fig. S3a, d).

The trimer interface of Eco2 RT domain is mediated by a loop region (residues 328‒342) primarily through hydrogen bonds involving amino acids D331, R332, and Y333 (Fig. 3b, c). Alanine substitutions of these residues revealed that R332A abolishes Eco2’s antiphage activity, while D331A and Y333A have no obvious effect (Fig. 3d). To confirm whether R332A affects trimer formation, we purified the R332A variant and compared it to the wild-type Eco2 complex using SEC. The R332A mutant partially impaired trimer assembly, indicating that this loop region alone is insufficient for trimer formation (Supplementary Fig. S4). Collectively, these results suggest that msDNA mediates the trimer formation of the Eco2 complex, while the RT-Toprim protein alone exists as a monomer.

### Retron-Eco2 RT domain forms extensive interactions with msDNA

As mentioned above, the base of the Eco2 trimer complex is a trimeric, triangular assembly of three RT domains and three msDNA molecules. The msrRNA portion of msDNA adopts an RSL flanked by 5′ and 3′ single-stranded tails. The RSL is sandwiched between RT_A_ and RT_B_ domains, with the 5’-end binding RT_A_ and the 3’-end binding RT_B_, respectively. This arrangement creates five extensive interfaces for RT‒msDNA interaction (Supplementary Fig. S5a). The 5’-end single-stranded tail primarily contacts the RT_A_ palm subdomain through hydrogen bonding and electrostatic interactions (Supplementary Fig. S5b). Notably, the rG15, which is crucial for reverse transcription initiation, forms hydrogen bonds with R184. The RSL region makes extensive contacts with the RT_A_ palm subdomain, as well as with Q329/R264 in RT_A_ thumb subdomain and RT_B_ thumb subdomain (Supplementary Fig. S5c, d). Although the Eco2 RSL loop sequence (rA30-rA31-rU32) differs from the loop in Eco1 (rU52-rU53-rU54), they share a similar conformation. In Eco2, rA30 and rU32 (Eco1 counterparts rU52 and rU54) flip outwards, while rA31 (Eco1 counterpart rU53) base-stacks with rG29 (Eco1 counterpart rG51) and aromatic residue H281 (Eco1 counterpart H268). These suggest that an aromatic residue (a conserved histidine) in the thumb subdomain facilitates the stabilization of the msrRNA loop. The ssRb region in the 3’-end tail is stabilized by interactions with residues F46 and R56 in the fingers subdomain, K245/R247/T249 in the palm subdomain, and S253 in the thumb subdomain of RT_B_ (Supplementary Fig. S5e). The DNA‒RNA duplex, closely aligned with the catalytic center YADD, engages in extensive interactions within the palm and thumb subdomains, stabilizing the catalytic pocket (Supplementary Fig. S5f).

### Insights into the reverse transcription process

**Fig. 4:**
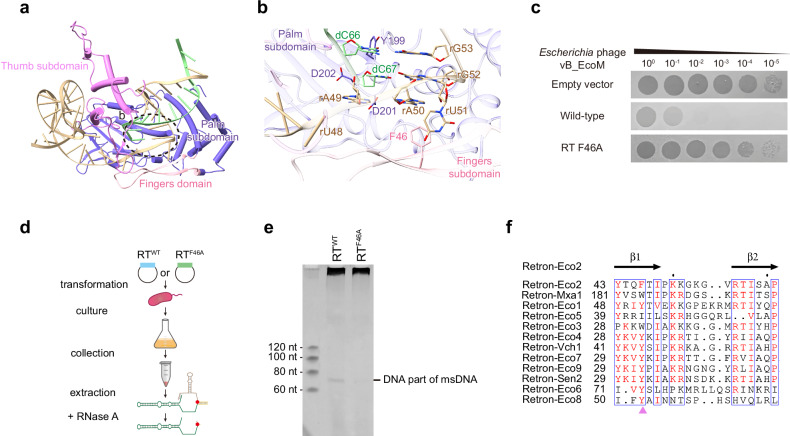
The conserved aromatic amino acid F46 in the fingers subdomain affects msDNA synthesis. **a** Overview of the Eco2 msDNA-RT domain, with the catalytic site outlined by a dashed ellipse. **b** Close-up view of the RT catalytic pocket, where rU51 flips out and stacks with the aromatic side chain of F46 (π–π interaction). **c** Serial dilution plaque assays with *Escherichia* phage vB_EcoM on *E. coli* MG1655, testing the wild-type vs RT^F46A^ mutant. The images are representative of three replicates. **d** Schematic flowchart showing the purification process of msdDNA portion of msDNA. **e** msDNA detection in RT^F46A^ mutant. 12% urea-PAGE analysis of msDNA extracted from cells harboring either wild-type Eco2 or RT^F46A^. The mutated system shows reduced msDNA production. **f** Alignment of RT domain sequences from various retrons, highlighting the conserved aromatic residue interacting with the terminal region of the msrRNA template.

Analysis of the msdDNA reveals that the last five nucleotides (dC63-dT64-dG65-dC66-dC67) match previous reports, reflecting a post-catalytic state (Fig. 2c, d). Y199 in the YADD motif forms a hydrogen bond network with the bases of dG65 and dC66 (which pair with rG53), while K66 and R70 interact with the phosphate backbone of msrRNA (Supplementary Fig. S5f). The last nucleotide, dC67, is positioned near the RT catalytic residues D201 and D202 (Fig. 4a, b). Interestingly, rU51 — poised as the next nucleotide to be reverse-transcribed — is flipped out and stabilized by π‒π stacking with an aromatic residue F46 in the fingers subdomain (Fig. 4b). Additionally, rA49 and rA50 stack with dC67 and rG52 (the template of dC67), respectively. Replacing F46 with alanine compromised antiphage activity (Fig. 4c) and reduced msDNA production in vivo (Fig. 4d, e), indicating that F46 is crucial for msDNA synthesis and defense.

Sequence alignment of diverse retron RTs shows this aromatic residue is highly conserved, often as a tyrosine (Fig. 4f). Analysis of Eco1 and Eco2 RTs structures reveals conserved YADD, NAXXH, VTG motifs and a similar arrangement of key residues (Supplementary Fig. S6a, b). In Eco1, the base of rU72 (Eco2 counterpart rU51) flips out and forms π‒π stacking interactions with dA76 and dG77 (Supplementary Fig. S6c). The Eco1 aromatic amino acid Y51 (Eco2 counterpart F46) forms a base-stacking interaction network with dA76, rU72, and dG77. Further antiphage assays showed that Y51A abolishes Eco1’s defense activity (Supplementary Fig. S6d). Together, these findings underscore the conserved role of this aromatic residue in msDNA synthesis and antiphage activity across retron RTs.

### Comparison of RT-msDNA complexes among retrons

The components of Eco2 differ significantly from those of Eco1, which belongs to the type II retron family, with a standalone nucleoside 2-deoxyribosyltransferase-like (NDT) effector^9,10^. In contrast, Eco2 integrates the RT and Toprim domains into a single protein. The overall structure of the Eco2 RT domain is similar to that of Eco1, with an RMSD of 3.3 Å over 288 residues. Interestingly, while Eco2 msrRNA forms a 23-bp RSL flanked by two single-stranded tails that bridge two RT domains, Eco1 msDNA adopts a roughly five-pointed star configuration wrapping around a standalone RT (Supplementary Fig. S7a, b). Several structural differences in the RT domains may explain their distinct oligomeric states and msDNA binding modes. For instance, the Eco2 α1 and α2 helices are antiparallel, whereas they are parallel in Eco1. In Eco1 the α1 helix mediates the dimer interface, tilting outward by ~13° compared to the Eco2 α1 helix (Supplementary Fig. S7c). Another difference is an inserted β sheet (β-sheet1) in the Eco2 palm domain that could hinder msrRNA binding if it adopted an Eco1-like conformation (Supplementary Fig. S7d, e). Additionally, Eco1 msrRNA has an extra RSL region absent in Eco2, potentially influencing whether the msrRNA encircles one RT or bridges two (Supplementary Fig. S7b, e). Finally, Eco2’s thumb domain features inserted β-sheet2, and a long loop that engages in mutual interactions among RT monomers in the trimer but do not solely dictate trimer formation (Supplementary Fig. S7f, g).

### Eco2 Toprim domain functions as an RNase to mediate antiphage activity

**Fig. 5:**
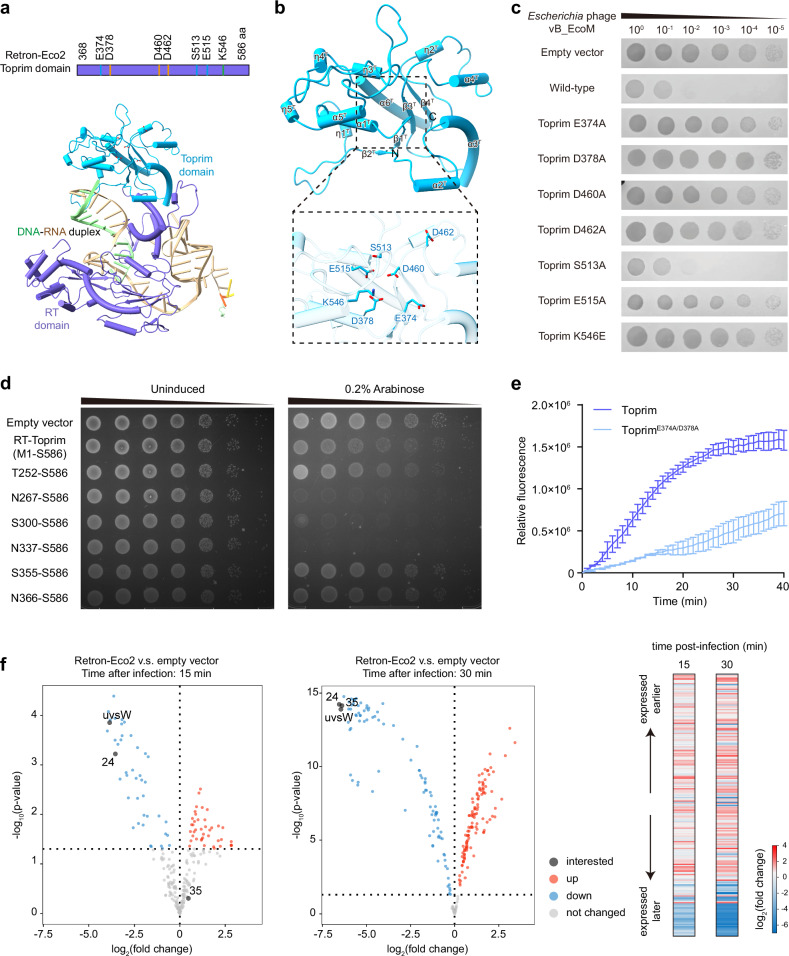
Functional Retron-Eco2 inhibits the expression of phage late genes. **a** Overall structure of Eco2 Toprim domain. Top panel: domain organization of the Eco2 Toprim domain, with key catalytic residues labeled. Bottom panel: cartoon representation of a single Eco2 protomer. **b** Detailed view of the catalytic pocket. Top panel: cartoon representation of the Toprim fold, with α-helices and β-strands labeled and “N”/“C” marking termini. Bottom panel: close-up of critical catalytic residues within the pocket. **c** Serial dilution plaque assays of *Escherichia* phage vB_EcoM on *E. coli* MG1655 expressing wild-type or Toprim-mutant Eco2 systems. The images are representative of three replicates. **d** Serial dilutions of *E. coli* MG1655 cells containing the pBAD-RT-Toprim and pBAD-Toprim plasmids on Luria-Bertani (LB) agar plates with or without 0.2% arabinose. Images are representative of three replicates. **e** RNase Alert assay of wild-type Toprim (N267‒S586) and corresponding active site mutant. Data are shown as the mean of three replicates with the standard error of the mean. **f** RNA-seq analysis of phage gene expression. Volcano plots (left, middle) and a heat map (right) illustrating differential phage gene expression in cells carrying Eco2 compared to an empty vector control.

Within the RT-Toprim fusion protein of Eco2, the C-terminal Toprim-like effector domain features a central four-stranded parallel β-sheet surrounded by α-helices (Fig. 5a, b). DALI analysis shows that the effector domain is structurally similar to class 1/2 OLD nuclease Toprim domains, as well as to effectors from the bacterial Gabija and PARIS antiphage defense systems^41,47,48,57^ (Supplementary Fig. S8). The OLD nuclease Toprim domain exhibits processive nuclease activity via a two-metal catalysis mechanism^57–61^. Consistent with this model, the Eco2 Toprim domain possesses a complete active site comprising an invariant glutamate, an invariant glycine, and a conserved DxD motif (Fig. 5b). Structural and sequence comparison of the Eco2 Toprim domain with the class 2 OLD nuclease (PDB: 6NK8) reveals conservation of residues E374/D460/D462 (DxD motif) coordinating metal A, D378/S513/E515 coordinating metal B, and the catalytic lysine K562 (Supplementary Fig. S9). Alanine substitutions in these residues abolished the antiphage activity of Eco2, indicating that Toprim enzymatic activity is crucial for its defense function (Fig. 5c).

To investigate the enzymatic activity of the Eco2 Toprim domain, we first assessed its potential host toxicity using RT-Toprim fusion protein and truncated Toprim domain constructs. Expression of the Toprim domain truncations (residues N267‒S586, S300‒S586, and N337‒S586) in *E. coli* resulted in pronounced growth inhibition, while the full-length RT-Toprim fusion protein exhibited no toxicity in spot growth assays (Fig. 5d). These results suggest that Toprim enzymatic activity is detrimental to the host and may contribute to Eco2-mediated antiphage defense.

To directly evaluate its catalytic function, we purified the Toprim domain truncation (residues N267‒S586) and assessed its activity using the RNase Alert assay. The wild-type Toprim domain displayed robust RNase activity, as evidenced by a time-dependent increase in fluorescence (Fig. 5e). In contrast, alanine substitutions in the predicted catalytic residues (E374 and D378) dramatically reduced RNase activity (Fig. 5e), indicating that the observed cleavage is specific and dependent on an intact active site.

We next performed RNA-seq analysis to determine the functional consequences of Eco2 activation during phage infection. Compared to the empty-vector control, Eco2-expressing cells displayed a marked downregulation of late phage genes and increased reads for early and middle genes at 15 min and 30 min post-infection (Fig. 5f). As successful progression to late gene expression depends on effective translation of earlier gene products, these data support a model in which Eco2 activation disrupts phage protein synthesis, likely via RNA degradation mediated by the Toprim domain.

Collectively, these findings reveal that the Retron-Eco2 Toprim domain functions as an RNase, and that this activity underlies its toxicity and Retron-Eco2’s antiphage function.

### Phage-encoded endonuclease IV-like protein DenB triggers Retron-Eco2 activation

The Toprim domain extends from the RT thumb subdomain and helps stabilize the DNA‒RNA duplex (Supplementary Fig. S10a). We also used AlphaFold2 to predict the structure of the isolated RT-Toprim protein. The model revealed a “closed” conformation compared to the more open arrangement observed by cryo-EM (Supplementary Fig. S10b, c). Within the resolved region of the protein, basic residues K383, H387, K418, R555, and K557 in the Toprim domain interact with the phosphate backbone of the RNA segment of the duplex (Supplementary Fig. S10d). To elucidate the functional state of Eco2, we superimposed its Toprim domain on that of DNA-bound GajA, a known active configuration of the Gabija defense system^41^. The key catalytic residues of Eco2 are aligned, yet we detect no Ca^2+^ density at the active site (Fig. 5b; Supplementary Fig. S10e). Moreover, the density for much of the msdDNA is absent, which could prevent substrate engagement and thus impede Toprim catalysis (Fig. S10f, g). Together with the observed RNase activity and toxicity of the isolated Toprim domain (Fig. 5d, e), these findings support a model in which Eco2 is maintained in an autoinhibited conformation and becomes activated only upon phage infection, likely through interactions with phage-derived factors.

**Fig. 6:**
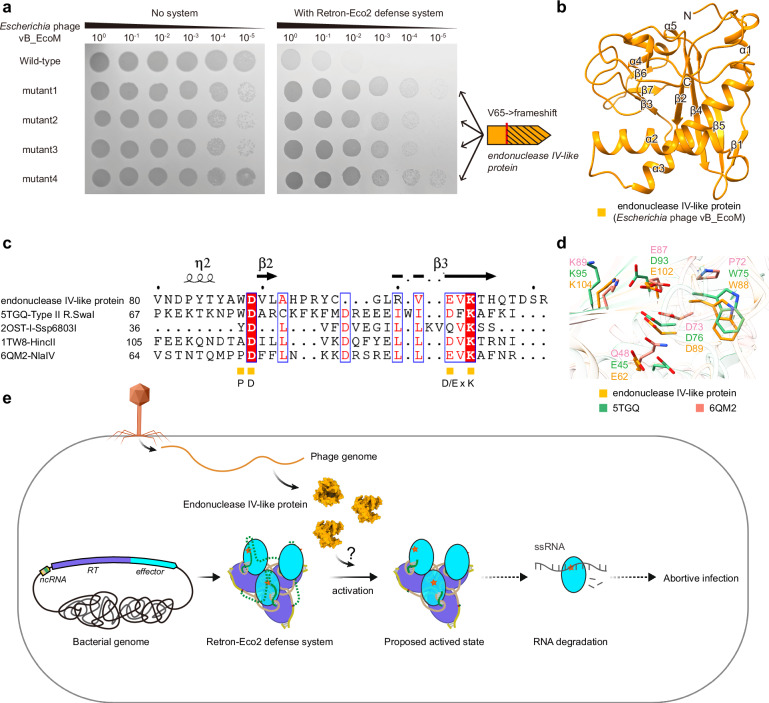
Phage endonuclease IV-like protein (DenB) triggers Retron-Eco2 activation and a proposed model for Eco2-mediated defense. **a** Phage mutants escaping Eco2 defense. Ten-fold serial dilution plaque assays with wild-type or mutant phages on *E. coli* MG1655 cells transformed with either an empty vector or the wild-type Eco2 system. Each mutant phage carries a thymine (dT) insertion between *denB* dT192 and dG193. This insertion results in a frameshift and the introduction of a premature stop codon. The images are representative of three replicates. **b** AlphaFold2-predicted structure of the DenB protein, an endonuclease IV-like enzyme putatively targeting ssDNA in a dC-specific manner. **c** Sequence alignment of DenB homologs. Secondary-structure elements are labeled above the alignment. Orange squares mark the conserved catalytic residues (PD_D/ExK). Alignment was performed with MultAlin. **d** Close-up view of the catalytic residues in DenB homologs, shown as sticks. **e** Proposed model for Eco2-mediated defense. Retron-Eco2 assembles into a self-inhibitory trimer, in which the msdDNA occludes the Toprim active site. Upon phage infection, DenB likely degrades msDNA, thereby relieving steric hindrance and enabling the Toprim domain to access and cleave RNA substrates, ultimately blocking phage replication.

To identify potential phage-encoded triggers of the Retron-Eco2 system, we isolated and sequenced phage mutants that evade the Eco2-mediated defense (escaper phages). Genome sequencing of four escapers revealed mutations in the *denB* gene, all featuring a thymine (dT) insertion between *denB* dT192 and dG193. This insertion results in a frameshift and the introduction of a premature stop codon (Fig. 6a). AlphaFold2-based predictions indicate that *denB* encodes an endonuclease IV-like protein that cleaves single-stranded DNA in a dC-specific manner^62,63^ (Fig. 6b). Sequence and structural alignments revealed that the DenB protein possesses a conserved ‘PD_D/ExK’ catalytic motif (Fig. 6c, d). The toxicity of DenB protein prevented direct assays to confirm its activation role. Nonetheless, the recurring *denB* mutations in escapers strongly suggest that the *denB* gene is integral to triggering Eco2 activation and that mutations disrupting its expression enable phages to circumvent this defense mechanism.

## Discussion

In this study, we show that Retron-Eco2 assembles into a supramolecular trimeric complex with a distinctive triangular quaternary architecture, distinct from previously reported retron-effector complexes^12,34,35,64^ (Fig. 1). Within this trimeric assembly, each msrRNA strand of msDNA bridges two RT-Toprim monomers. Notably, the formation of a specific RSL region in msrRNA appears critical for mediating these interactions (Supplementary Fig. S5). Functionally, we show that the Toprim domain of Eco2 exhibits RNase activity, providing a mechanistic basis for its role in antiphage defense. Furthermore, we identified the phage-encoded DenB protein as a potential trigger of Eco2 activation. Together, these findings reveal a new structural principle underlying Retron-Eco2-mediated defense and highlight how diverse retron systems may adopt distinct conformations to combat phage invasion.

A central feature of Eco2 is the Toprim effector domain, which shares a conserved core fold with other bacterial immune effectors^39,41–44,47,48,65–67^ (Supplementary Fig. S8). The functional diversification of Toprim domains in bacterial immunity underscores the evolutionary plasticity of this fold^7,60,61^. For instance, Gabija cleaves double-stranded DNA (dsDNA), Wadjet (JET) targets plasmid DNA, and PARIS disrupts host tRNA^Lys^ to stall translation^38,42,47^. Our biochemical assays and RNA-seq analysis suggest that the Eco2 Toprim domain functions as an RNase upon activation, targeting and degrading RNA to arrest phage development prior to the late stages of infection (Fig. 5). Our data, as well as another study^65^, further implicate the phage-encoded endonuclease IV-like protein DenB as a trigger for Retron-Eco2 activation. We propose that, under normal conditions, the flexible msdDNA region occludes the Toprim active site (Supplementary Fig. S10f, g), maintaining the complex in an autoinhibited conformation (Fig. 6e). During phage infection, DenB likely degrades the msdDNA scaffold, thereby relieving steric hindrance and enabling the Toprim domain to access and cleave RNA substrates, ultimately blocking phage replication (Fig. 6e).

From a biotechnology standpoint, retrons offer unique advantages because they naturally generate multiple copies of single-stranded DNA. This property has been harnessed for donor DNA production in genome editing applications across diverse organisms^21,23,25,26,30,68–76^. Our work provides the first detailed blueprint for Eco2’s trimeric assembly, setting the stage for structure-guided optimization of retron-based editing tools and novel phage therapies. As these genome engineering approaches grow in scope and sophistication, refining our structural and mechanistic understanding of retrons like Eco2 will be essential for advancing their design.

## Materials and Methods

### Bacteria and phage strains

*Escherichia coli* strains [MG1655, DH5α, and BL21(DE3)] were cultured in LB or on LB agar at 37 °C with shaking at 210 rpm, unless otherwise stated. To maintain plasmid stability, the media were supplemented with ampicillin (100 μg/mL), kanamycin (50 μg/mL), or chloromycetin (25 μg/mL), depending on the resistance conferred by the plasmids. Phage infections were performed in LB medium supplemented with 0.1 mM MnCl_2_ and 5 mM MgCl_2_ (MMB medium) at 37 °C. Unless otherwise indicated, 0.2 mM isopropyl-β-d-thiogalactopyranoside (IPTG) was used for induction.

### Plasmid construction

The Retron-Eco2 genetic unit consists of a native promoter, ncRNA, and an RT-Toprim fusion protein. The genes encoding for native promoter and ncRNA, which are not codon-optimized, as well as the full-length RT-Toprim protein, which is codon-optimized for expression in *E. coli*, were synthesized by General Biosystems (Anhui) Co., Ltd. For protein expression, purification, and structural studies, the ncRNA was introduced into pBB75 vector (Novagen), while the coding sequence for RT-Toprim protein was cloned under the control of a T7 promoter into the pET21b vector, which includes a C-terminal 8× His tag (His8). For phage challenge assays, the Retron-Eco2 genetic unit was assembled through gene splicing via overlap extension PCR and subsequently subcloned into pACYC184 vectors. The mutant Eco2 genetic units were generated using the original sequence as a template.

### Phylogenetic analysis

A phylogenetic tree of retron RTs was constructed using sequences from Mestre et al.^10^ and Bobonis et al.^11^ Sequences with an identity greater than 99% were eliminated using Mmseqs2^77^ (v.13.45111). The multiple sequence alignment employed to generate the tree was performed using MUSCLE v.5.1^78^ with the super5 model, followed by trimming with trimAL v.1.5.0^79^ using the automated1 option. Finally, the retron RTs tree was constructed using IQ-TREE v.2.3.2^80^ with ModelFinder and Ultrafast Bootstrap set to 1000.

### Expression and purification of Retron-Eco2

For the protein expression of the Retron-Eco2 complex and its mutants, the pET21b-rrt-toprim-His8 and pBB75-ncRNA constructs were co-transformed into the *E. coli* BL21 (DE3) strain, plated on LB agar plates, and grown overnight at 37 °C. A single colony was inoculated into 10 mL of LB medium supplemented with 100 μg/mL ampicillin and 50 μg/mL kanamycin as starter culture, and grown at 37 °C with shaking overnight. The 10 mL starter culture was then added to 1 L of LB medium (supplemented with 100 μg/mL ampicillin and 50 μg/mL kanamycin) and incubated at 37 °C with shaking at 200 rpm until reaching an OD600 of 1.0. The cultures were subjected to cold shock at 16 °C for 30 min and then induced with 200 μM IPTG. Following induction, the cultures were incubated for a further 16 h. Cells were pelleted and resuspended in lysis Buffer containing 25 mM Tris-HCl, pH 8.0, 150 mM NaCl, and subsequently lysed using a pressure homogenizer at 4 °C. Cellular debris was removed by centrifugation at 20,000× *g* for 1 h at 4 °C. The supernatant was loaded onto a column containing Ni^2+^ affinity resin (Ni-NTA, Qiagen), and the protein complex was eluted using buffer E (25 mM Tris-HCl, pH 8.0, 250 mM imidazole). The eluate was applied to a Source15Q column (GE Healthcare) and eluted using a NaCl gradient from 0 to 1 M in 25 mM Tris-HCl, pH 8.0 and 2 mM 1,4-dithiothreitol (DTT). The fractions corresponding to a conductivity of approximately 35 mS/cm were concentrated to 1 mL and subsequently purified by gel filtration chromatography (Superose6 10/300, GE Healthcare) in buffer SD (25 mM Tris-HCl, pH 8.0, 150 mM NaCl and 5 mM DTT).

The RT-Toprim-His8 apo protein, the Toprim domain (N267-S586) and corresponding mutants were expressed from pET21b plasmid in the *E. coli* BL21(DE3) strain. With the exception of the addition of 100 μg/mL ampicillin during cell culture, all other conditions were consistent with those mentioned previously. For protein purification, cell pellets were resuspended in high-salt lysis Buffer 2 (25 mM Tris-HCl, pH 8.0, 500 mM NaCl), followed by cell lysis and centrifugation to remove cell debris. The supernatant was incubated with Ni^2+^ affinity resin (Ni-NTA, Qiagen) that had been pre-equilibrated with Lysis Buffer. The target protein was eluted by Lysis Buffer 2 supplemented with 250 mM imidazole. The eluted sample was applied to a Heparin column (GE Healthcare) and subjected to an increasing gradient NaCl elution from 500 mM to 2 M NaCl in 25 mM Tris-HCl, pH 8.0 and 2 mM DTT. The fractions containing the target protein were concentrated to 1 mL. The RT-Toprim-His8 apo protein was further purified by gel filtration chromatography (Superose6 10/300, GE Healthcare) in buffer SD (25 mM Tris-HCl, pH 8.0, 350 mM NaCl) supplemented with 5 mM DTT, while the Toprim domain (N267-S586) and its mutants further purified by gel filtration chromatography (Superdex 200 10/300, GE Healthcare) with the same buffer as the RT-Toprim-His8 apo protein.

For the Retron-Eco2 complex and RT-Toprim apo protein, UV absorbance values were measured at 260 nm and 280 nm. The final purified proteins were analyzed by SDS-polyacrylamide gel electrophoresis (SDS-PAGE) and 8% -urea-PAGE gel.

### Cryo-EM grid preparation and data acquisition

4 µL aliquots of the freshly purified Retron-Eco2 complex at an approximate concentration of 1.0 mg/mL were applied to glow-discharged Quantifoil Au R1.2/1.3 (300 mesh). The grids were plunge-frozen in liquid ethane after blotting for 4.5 s at 100% humidity and 8 °C using a Vitrobot (Mark IV, Thermo Fisher Scientific). The cryo-grids were then transferred to 300 kV Titan Krios electron microscopes (Thermo Fisher) equipped with a GIF Quantum energy filter (slit width 20 eV) and a Gatan K3 Summit detector. Each image was automatically collected using the EPU software (version 2.9, Thermo Fisher Scientific) at a magnification of 81,000×, resulting in a pixel size of 1.07 Å/pix. Each micrograph stack, containing 32 frames, was exposed for 3.5 s. The defocus range was between ‒1.2 and ‒2.2 μm with an accumulated total dose of 50 e^‒^/Å^2^ and determined by Gctf.

### Cryo-EM data processing

A diagram illustrating the procedures for data processing is presented in Supplementary Fig. S2. A total of 5092 movie stacks were motion-corrected using MotionCor2, and the resulting micrographs were subjected to Patch CTF estimation and correction in cryoSPARC^81^. After manual inspection and curation, 4098 micrographs were selected for further processing. Particles were automatically picked using blob-picker in combination with Topaz, and 3,108,650 particles were extracted with a box size of 240 pixels, followed by Fourier cropping to 60 pixels. Subsequent 2D classification was performed, and 41,623 particles with distinct features were selected for ab initio model generation, resulting in the creation of 10 initial models, including 1-class, 3-class, and 6-class models. These models were used as multi-references for 3D classification. A total of 2,979,419 particles were subjected to two rounds of 3D classification, yielding 1,307,521 high-quality particles, which were re-extracted with a box size of 240 pixels and subjected to a third round of 3D classification. Finally, a subset of 574,158 particles was selected for 3D refinement, resulting in a cryo-EM map with an overall resolution of 2.6 Å based on gold-standard Fourier shell correlation (FSC)^82^.

### Model building and refinement

For the atomic model of the Retron-Eco2, the Alphafold2 predicted structure was fitted into the cryo-EM density map using the “fit in the map” function in UCSF ChimeraX^83^. The initial fitted model was further refined through manual adjustments in COOT v0.8.9.1^84^. The resulting protein model was subjected to a rigid fit into the the entire cryo-EM density map via the phenix.dock_in_map function of the PHENIX v1.21 software suite^85^. Model quality was assessed using the Molprobity scores^86^. Finally, structural figures were generated via ChimeraX (v.1.2.5).

### Plaquing assays

Plaque assays were performed as previously described^3,9,87^. In brief, *E. coli* MG1655 containing the retron system or the pACYC184 empty vector was cultured. Bacterial lawns were prepared by mixing 200 μL of overnight cultures with 7 mL of molten top agar supplemented with 25 μg/mL chloromycetin, which was then poured onto LB agar plates. Tenfold serial dilutions of a high-titre phage stock were spotted onto the top bacterial layer. The plates were imaged after overnight incubation at 37 °C. The efficiency of plating (EOP) was measured and compared between the plaque assay results for control bacteria and those containing the defense system. The activity of wild-type Retron-Eco2 system and its mutants were assessed by performing EOP assays with *Escherichia* phage vB_EcoM, while retron Eco1 system and its mutants were infected with phage K42.

### Isolation of escaper bacteriophages that overcome defense

To isolate mutant bacteriophages that escape the Retron-Eco2 defense system, phages were plated on LB agar plates containing bacteria expressing the wild-type Retron-Eco2 system using a double-layer plaque assay^9,87^. Overnight cultures of *E. coli* MG1655 containing the Retron-Eco2 defense system were diluted 1:50 and grown at 37 °C with shaking for 1.5 h until reaching an OD600 of 0.3. 200 μL of the culture was mixed with 7 mL of pre-melted 0.5% MMB agar containing chloromycetin and poured onto agar plates. 2 μL drops of tenfold serial diluted bacteriophage vB_EcoM were spotted onto the bacterial top layer. The double-layer plates were incubated overnight at 37 °C, and single plaques were picked into 90 μL of phage buffer (50 mM Tris, pH 7.4, 100 mM MgCl_2_, 10 mM NaCl). The phage solutions were mixed several times by vortexing to release the phages from the agar into the phage buffer, and then centrifuged at 2000× *g* for 10 min to pellet debris. The supernatant containing the mutant phages was sterile-filtered and transferred into a new tube. The collected phages were tested for their ability to infect both a control strain and a Retron-Eco2 operon-carrying strain, and their phenotype was checked and compared to that of the parental bacteriophage vB_EcoM through plaque assays, as described above.

### Amplification of phages

Amplification of phages was performed as previously described^9,87^. Parental bacteriophages were propagated in *E. coli* MG1655 containing an empty vector, while escaper phages were more effectively propagated on the Retron-Eco2 operon-carrying strain. The *Escherichia* phage vB_EcoM was propagated in a liquid culture of bacteria grown in MMB medium to an OD600 of 0.3. After incubation for 3 h at 37 °C with shaking at 200 rpm, the lysate was centrifuged for 10 min at 2000× *g*, and the supernatant was filtered through a 0.22 μM filter to remove remaining bacterial cell debris. The phage titer was determined using the plaquing assay.

### msDNA isolation

msDNA isolation was performed as described previously^9,11^. Briefly, 25 mL cultures of *E. coli* MG1655 expressing wild-type Retron-Eco2 defense system and its mutant from the native promoter were grown in LB medium supplemented with chloromycetin at 37 °C with shaking at 200 rpm. Overnight cultures were centrifuged at 2000× *g* for 10 min. The supernatants were discarded, and the cell pellets were resuspended in 200 μL of Solution I (50 mM glucose, 10 mM EDTA and 25 mM Tris-HCl, pH 8.0). Next, 200 μL of Solution II (0.2 N NaOH, 1% SDS) was added, and the mixture was inverted gently ten times. Subsequently, 400 μL of Solution III (3 M potassium acetate, 2 M acetic acid) was added, and the samples were mixed again by inverting the tubes five times. After centrifugation, the supernatant was precipitated overnight at ‒80 °C with ethanol. The precipitated nucleic acids were centrifuged at 12,000× *g* for 30 min at 4 °C, and the supernatant was discarded. The pellet was washed twice with 1 mL of 70% ethanol and centrifuged at 12,000× *g* for 10 min at 4 °C. The pellets were air-dried for 15–30 min at room temperature and resuspended in 20 μL diethyl pyrocarbonate (DEPC)-treated water. The purified nucleic acids containing msDNA were treated with RNase A for 30 min at room temperature. The samples were then incubated with loading buffer (90% formamide, 20 mM Tris-HCl, pH 8.0, 20 mM EDTA, pH 8.0, 0.05% (w/v) bromophenol blue 0.05% (w/v) xylene cyanol) at 100 °C for 5 min and subjected to 12% denaturing Urea-PAGE using 1× Tris/Borate/EDTA buffer (150 V, 1 h). The urea-PAGE gels were strained with 4S GelRed.

### RNase alert assay

To detect the RNase activity of Toprim domain, we used RNase Alert kit (APExBIO, Cat. No. K1902). The RNase Alert kit, based on the principle of Fluorescence Resonance Energy Transfer (FRET), is capable of detecting RNase activity with high sensitivity. We co-incubated the purified Toprim domain with ssRNA provided in the kit at 37 °C and performed fluorescence detection using SpectraMax^®^ i3x, with an excitation wavelength of 490 nm and an emission wavelength of 520 nm. Culves were analyzed and generated using GraphPad Prism v.8.0.2.

### Detection of the phage genes expression using RNAseq

For each sample, *E. coli* MG1655 cells containing the Retron-Eco2 defense system or the pACYC184 empty vector were grown in 10 mL of LB medium at 37 °C with shaking at 200 rpm until reaching an OD600 of 0.3. The cultures were infected with *Escherichia* phage vB_EcoM at a multiplicity of infection (MOI) of 5. RNA was extracted from the cells using Bacterial Total RNA Rapid Extraction Kit (Sangon Biotech, Order NO. B518625) at 15 min and 30 min post-infection, as previously described. Briefly, cells were collected by centrifugation at 12,000× *g* for 1 min at 4 °C. The pellets were incubated with 100 μL of lysozyme (400 μg/mL) for 5 min at room temperature. Next, 900 μL of Buffer Rlysis-B was added, and the mixture was vortexed immediately before incubated for 5 min at room temperature. Subsequently, 200 μL of chloroform was added, and the tubes were mixed. The samples were centrifuged at 12,000× *g* for 5 min at 4 °C. The supernatant was precipitated with one-third volume of ethanol for 3 min at room temperature and then centrifuged at 12,000× *g* for 5 min at 4 °C. The pellets were washed twice with 750 μL of 75% ethanol, centrifuged again at 14,000 rpm for 3 min at 4 °C, air-dried for 10 min, resuspended in 30–50 μL of DEPC-treated ddH_2_O, and stored at –80 °C. All the experiments were performed with two biological replicates. The extracted RNA was sent to Seqhealth Technology Co., Ltd (Wuhan, China) for subsequent cDNA library preparation, and high-throughput sequencing using Illumina sequencing platform (Novaseq 6000). Differentially expressed genes were identified using EdgeR based on pairwise comparison (the criteria BH-adjusted *P* < 0.05 and |log2 Fold Change | > 1).

### Quantification and statistical analysis

Details of specific statistical tests are provided in the figure legends. Data represent the mean of two biological replicates, unless stated otherwise.

## Supplementary information

Supplemental information

## Data Availability

The atomic coordinates and EM density for the reported structures of the Retron-Eco2 complex (PDB: 9LM3; EMDB: EMD-63214) have been deposited in the Protein Data Bank (www.rcsb.org) and the Electron Microscopy Data Bank (www.ebi.ac.uk/pdbe/emdb/). All raw genome sequencing data for the wild-type and four mutant phage *Escherichia* phage vB_EcoM, and raw RNA-seq data have been deposited under NCBI Bioproject PRJNA1212231.
