# Supplementary material for: Structural basis of the RNA-mediated Retron-Eco2 oligomerization
Source: Cell Discov. 2025 Sep 2;11:73. doi: 10.1038/s41421-025-00823-y (PMC12405507; doi:10.1038/s41421-025-00823-y)
Supplement: Supplementary file 1 — Supplemental information [file 41421_2025_823_MOESM1_ESM.pdf]

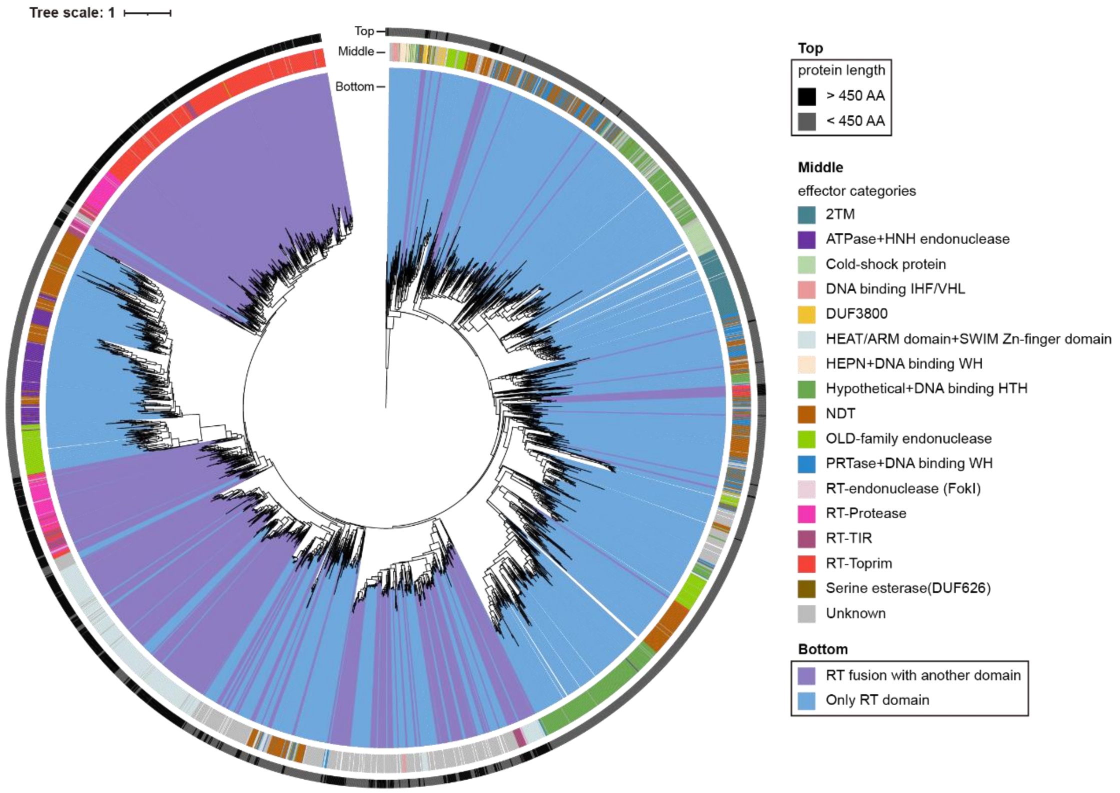

Supplementary information, Fig. S1 Phylogeny of retron RTs. Phylogenetic tree highlighting the evolutionary relationships among retron RT homologs.

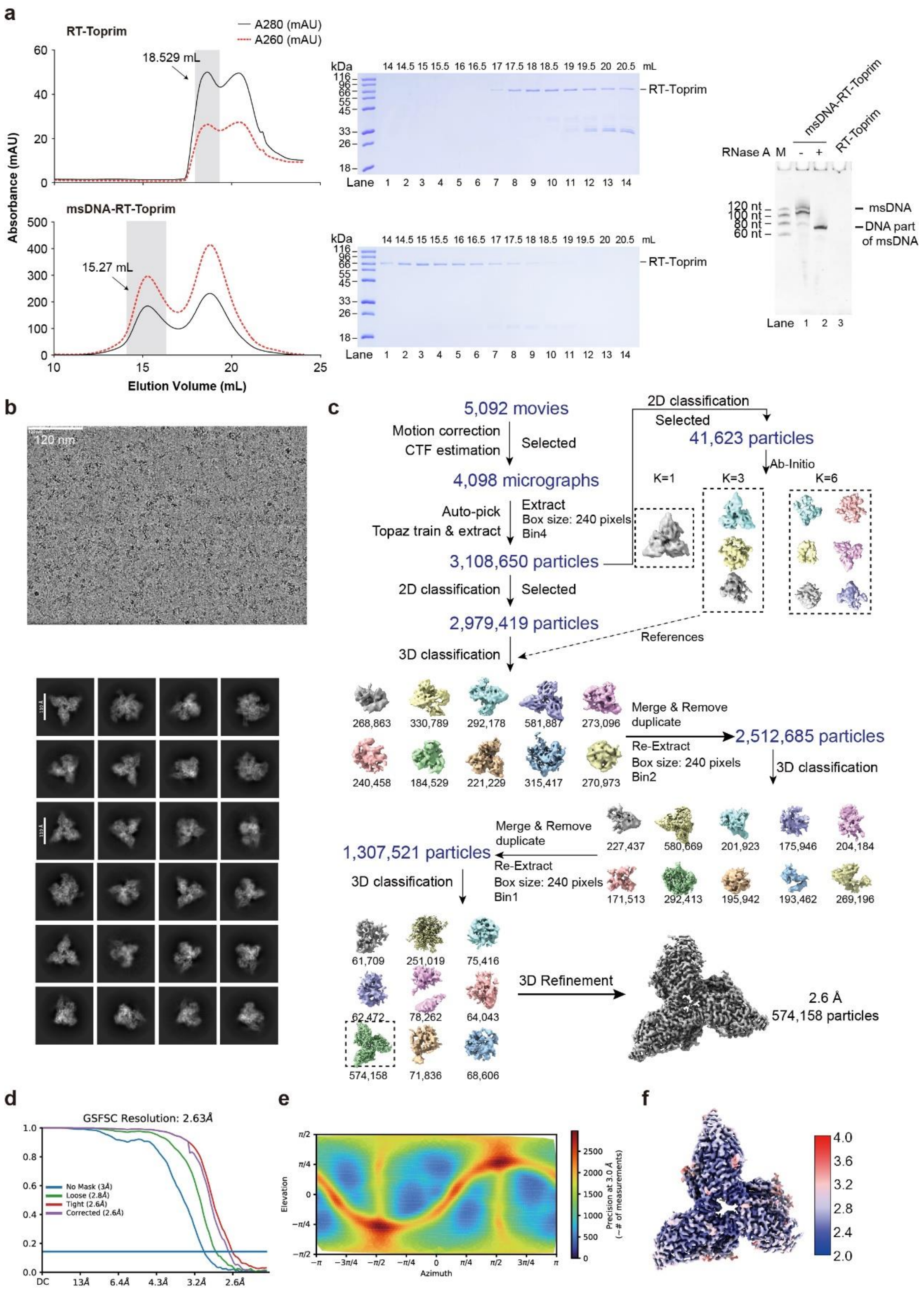

Supplementary information, Fig. S2 Cryo-EM data processing for the Retron-Eco2 complex. a Gel filtration chromatography, 15% SDS-PAGE, and 12% urea–PAGE analyses showing the purification of the RT-Toprim apo protein and the msDNA-bound Eco2 complex. Fractions containing the Eco2 complex (elution volume ~14–16 mL) were used for cryo-EM sample preparation. **b** Representative micrograph (from 5,092 micrograph dataset) and 2D classes displaying the Retron-Eco2 complex.

Scale bar = 120 nm. **c** Workflow outlining the cryo-EM reconstruction process for *E. coli* Retron-Eco2 complex. **d** Gold standard FSC curve illustrating the final global refinement of Retron-Eco2. **e** Angular distribution for the final reconstruction. **f** The local map resolutions of the final structure.

a

	Chain	Z	rmsd	Description
→	7V9X-A	26.3	3	Retron-Eco1 RT
→	9C0I-A	19.2	4.5	DRT2 RT
→	7K9Y-A	18	4.1	Group II intron GsI-IIC RT Trt
	8BGJ-A	15.9	2.8	CART-CAPP from *Caloramator australicus*
→	8UW3-A	15	4.9	LINE-1 retrotransposable element ORF2 protein
	8OZ7-A	14	3.7	*Ll*AbiA
	8H2H-D	13.6	3.3	Group II intron-encoded RT LtrA
→	8IBW-C	13.2	3.9	R2 retrotransposon
	6ZDU-A	12.5	4.4	Telomerase RT
	8FLI-B	12	4.6	Group II intron maturase RT
	7OJN-L	11.2	3.6	Lassa virus L protein RNA-directed RNA polymerase (RdRp)
	7SR6-A	11.1	3.5	HERV-K RT
→	3DU5-A	11	4.3	Telomerase TERT
→	7R08-E	10.9	4.4	Abi-P2
	8PSN-B	10.8	4.4	Polymerase acidic protein (PA-like)
	7R07-F	10.8	4	AbiK
	7EL9-A	10.6	3.8	RdRp of Machupo virus L protein
	7ELB-A	10.4	3.6	RdRp of Machupo virus L protein
	6Y6K-A	10.1	4	RdRp of Phenuiviridae L protein
	8H69-B	10.1	4.8	Influenza RNA polymerase acidic protein

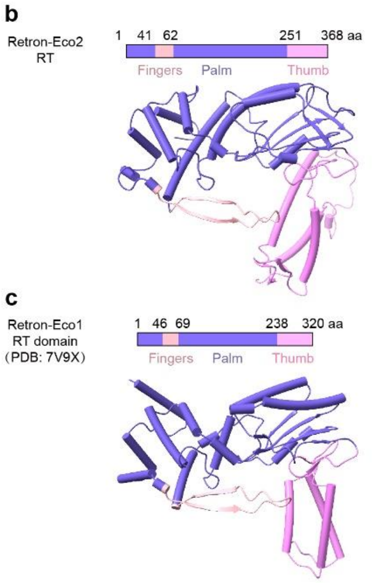

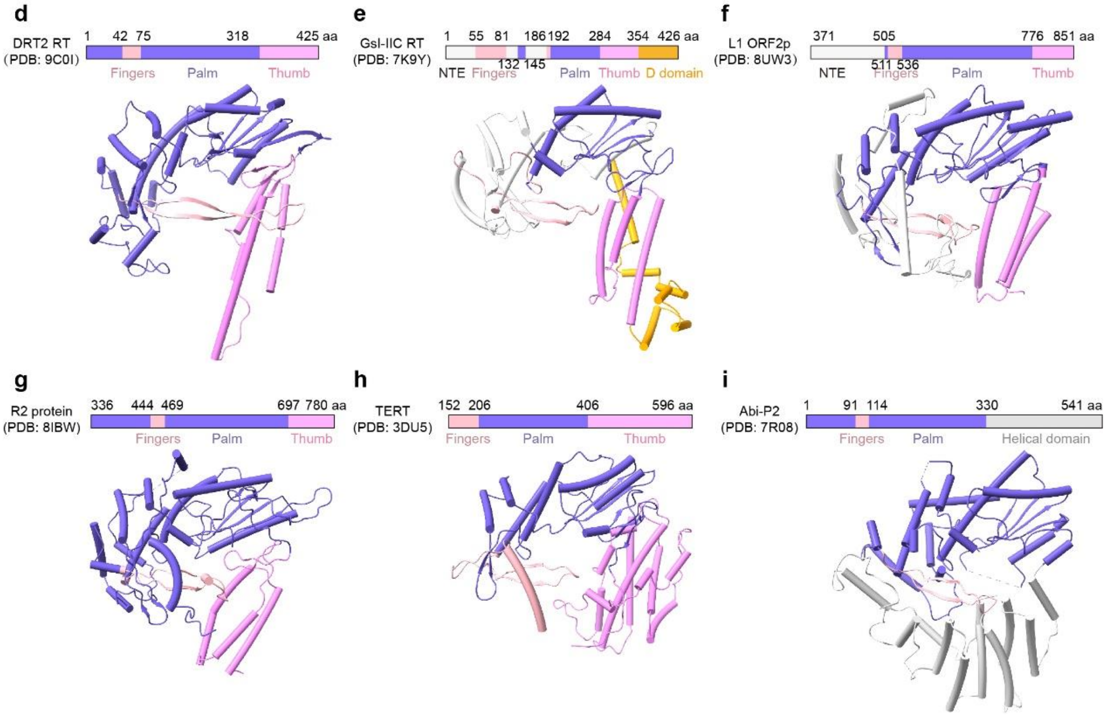

Supplementary information, Fig. S3 Comparison of RTs across different domains of life. a Table of high-scoring Eco2 RT structural homologs in DALI analysis. (**b-i**) Cartoon views of selected RT structures, color-coded as follows: NTE in white, D domain in orange, C-terminal helical domain in dark gray, fingers in light pink, palm in slate blue, thumb in violet. **b** RT domain of Retron-Eco2. **c** Cryo-EM structure of *E. coli* Retron-Eco1 in complex with its effector (PDB: 7V9X, residues: 1-320). **d** Cyro-EM structure of the DRT2 RT in complex with its non-coding RNA (PDB: 9C0I, residues: 1-425). **e** Crystal structure of a template-swicthing (TS) complex of a group II intron RT (PDB: 7K9Y, residues: 1-426). **f** Cryo-EM structures of the complete human L1 (long interspersed element-1, LINE-1) ORF2p (PDB: 8UW3, residues: 371-

851). **g** Cryo-EM structure of eukaryotic R2 retrotransposon from *Bombyx mori* (PDB: 8IBW, residues: 336-780). **h** Crystal structure of catalytic subunit of telomerase (TERT) from *Tribolium castaneum* (PDB: 3DU5, residues: 152-596). **i** Crystal structure of *E. coli* Abi-P2 (PDB: 7R08, residues: 1-541).

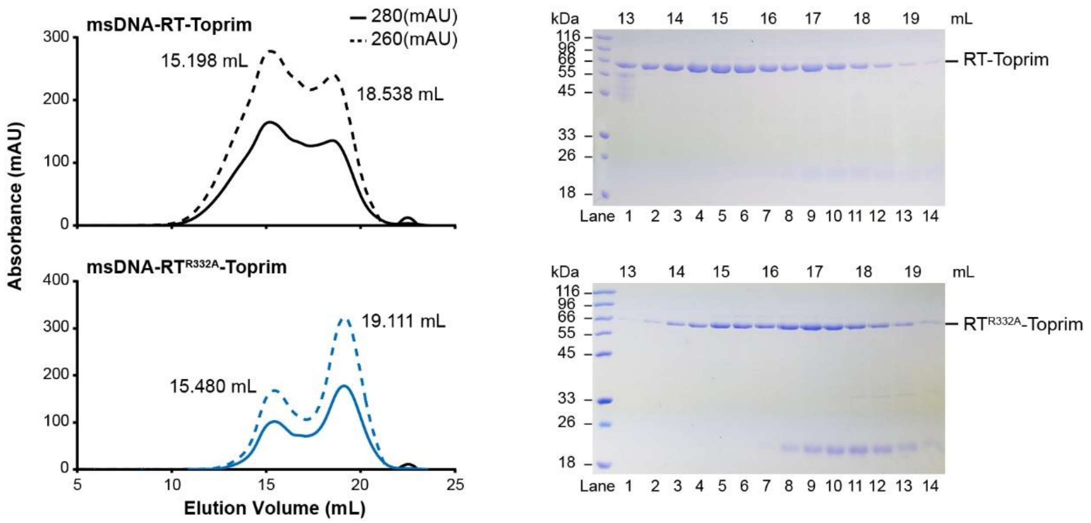

Supplementary information, Fig. S4 Purification of the Retron-Eco2 defense complexes. Size exclusion chromatograms of Eco2 msDNA-RT^{WT}-Toprim and msDNA-RT^{R332A}-Toprim complexes, showing absorbance at A280 (protein) and A260 (nucleic acid). Peak fractions were evaluated by SDS-PAGE to confirm complex formation.

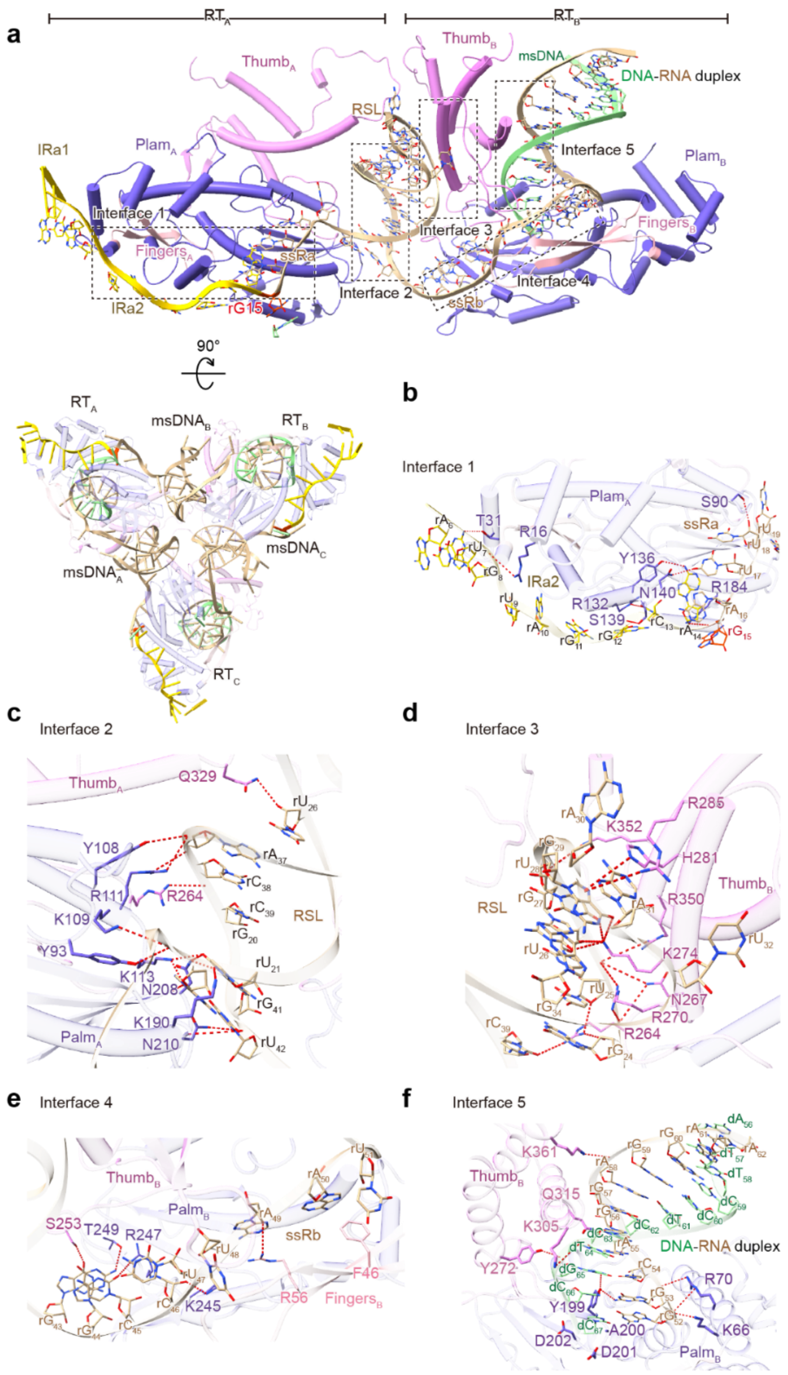

Supplementary information, Fig. S5 Detailed intermolecular interactions between Eco2 msDNA and RT. a Cartoon representation of msrRNA bound to adjacent RT domains (RT_A and RT_B). **b-f** Close-up views of Eco2 msrRNA-RT interactions. Panels show interactions of IRa2 and ssRa with RT_A (**b**), RSL-RT_A (**c**), RSL-RT_B (**d**), ssRb-RT_B (**e**), and the DNA-RNA duplex with RT_B (**f**). The msrRNA portion is in tan, msdDNA in light green, IRs in gold, rG15 in orange-red, the branching G (rG15) is highlighted in orange-red, and the Toprim domain in deep sky blue. The RT domain is depicted in matching colors: the palm in slate blue, the fingers in light pink, and the thumb in violet.

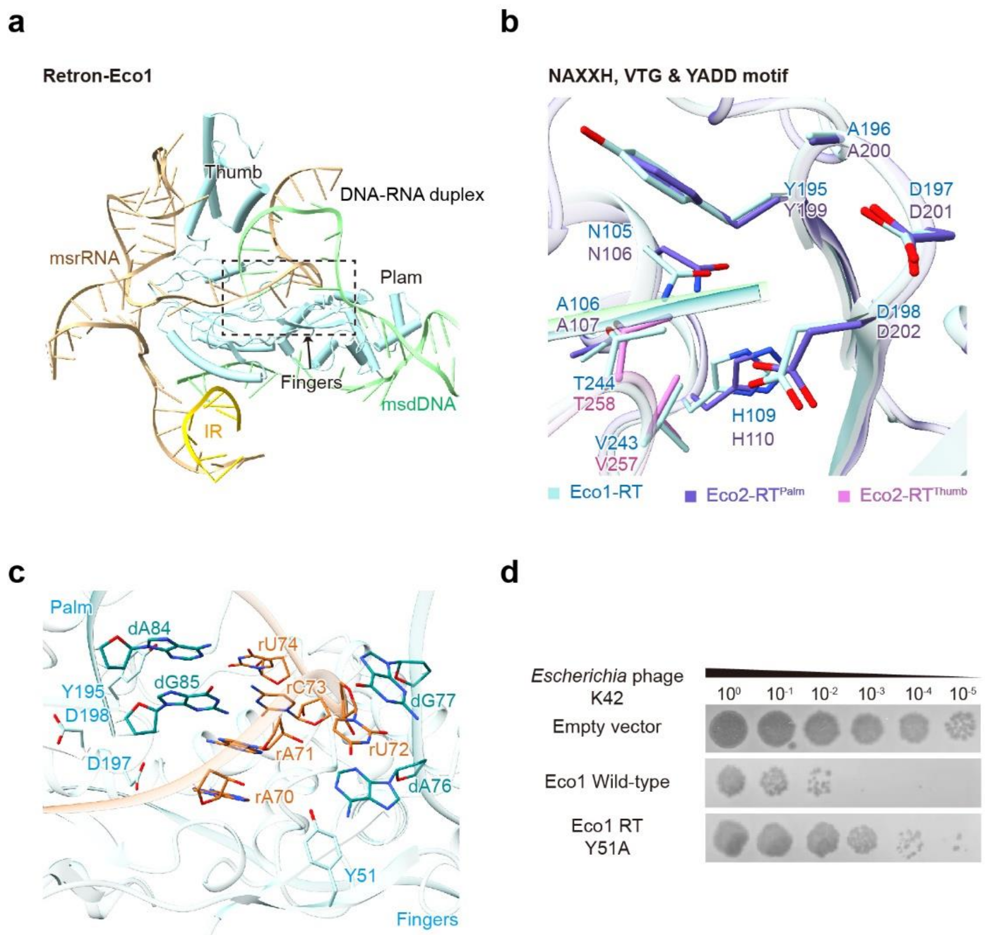

Supplementary information, Fig. S6 Conserved active sites and aromatic amino acid in Retron-Eco1. a Eco1 msDNA-RT protomer. Cartoon view with RT in pale turquoise, msdDNA in dark cyan, msrRNA in chocolate, IR in orange, rG14 in dark red. **b** Conserved X and Y motifs. Comparison of Eco2 and Eco1 RT domains reveals conserved segments (NAxxH and VTG) near the catalytic core. **c** Close-up view of Eco1 active site showing rU72 flipped out and stabilized by Y51 in the finger subdomain. **d** Serial dilution plaque assays testing wild-type or mutant Eco1 in *E. coli* MG1655 against *Escherichia* phage K42.

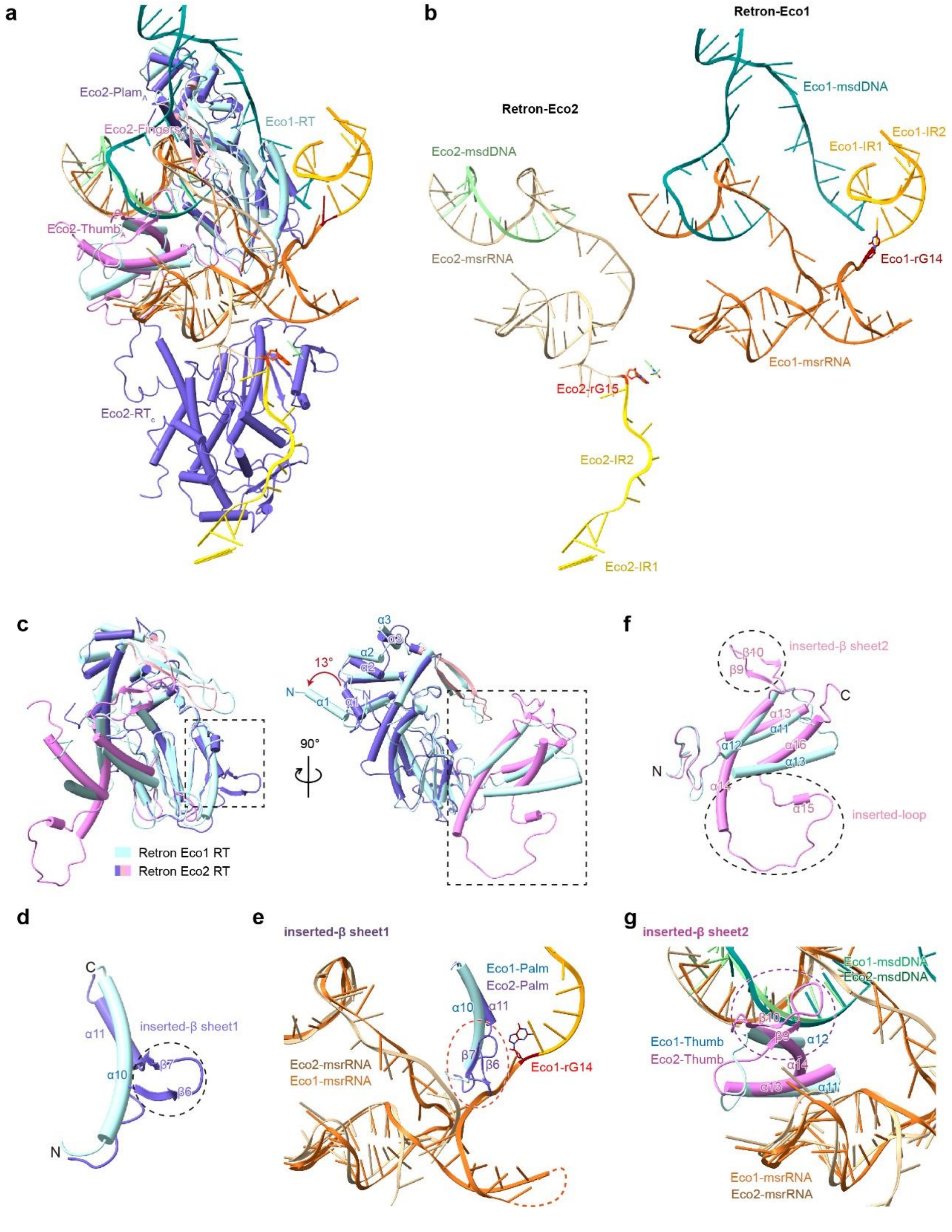

Supplementary information, Fig. S7 Distinct binding modes of msDNA to RT in Eco2 and Eco1. a Superposition of the msDNA-RT complexes from Eco2 and Eco1 (PDB: 9JM0; RMSD 3.28 Å over 288 residues). The finger, palm, and thumb subdomains of the Eco2 RT domain are depicted in light pink, slate blue, and violet, respectively, while the Eco1 RT protein is colored pale turquoise. **b** The atomic structures of Eco2 msDNA and Eco1 msDNA. The Eco2 msdDNA, msrRNA, IR, rG15 are shown in light green, tan, gold, and orange red, respectively. In contrast, the Eco1 msdDNA, msrRNA, inverted repeat (IR), rG14 are colored dark cyan, chocolate,

orange, and dark red, respectively. **c** Superposition of the RT domains from Retron-Eco2 and Eco1. The colors of Eco2 RT domain are described as previously mentioned, while the Eco1 RT is colored dark cyan. The main differences are highlighted within a dashed box. **d** A notable difference in the palm subdomain is the presence of an additional inserted β-sheet1 in Eco2, which is absent in Eco1. **e** The α11 helix in Eco2 is shorter than the corresponding α10 helix in Eco1. Additionally, Eco2 possesses an inserted β-sheet2 within the palm subdomain that spatially clashes with the msrRNA of Eco1. **f** Two distinct features in the thumb subdomains of the Eco2 and Eco1 RT domains are highlighted: the inserted β-sheet2 and an inserted loop, both of which are unique to Eco2. **g** The inserted β-sheet1 of the Eco2 thumb subdomain, which is absent in Eco1 RT, interacts with the DNA part of the DNA-RNA hybrid.

a

Chain	Z	rmsd	Description
6NJW-A	7.7	4.1	Class 2 OLD family nucleases from *Xanthamonas campestris p.v. campestris*
8X5I-A	7	3.6	Endonuclease GajA of Gabija defense system
2P62-A	6.5	5.1	Hypothetical protein PH0156 from *Pyrococcus horikoshii* OT3
8UX9-A	6	3.7	AriB of the PARIS immune complex
6P74-A	5.2	3.7	Class 1 OLD family nucleases from *Thermus Scotoductus*
6OAP-A	5.2	3.7	A dual sensor histidine kinase
2FCJ-B	4.9	2.7	Small TOPRIM domain protein from *Bacillus stearothermophilus*

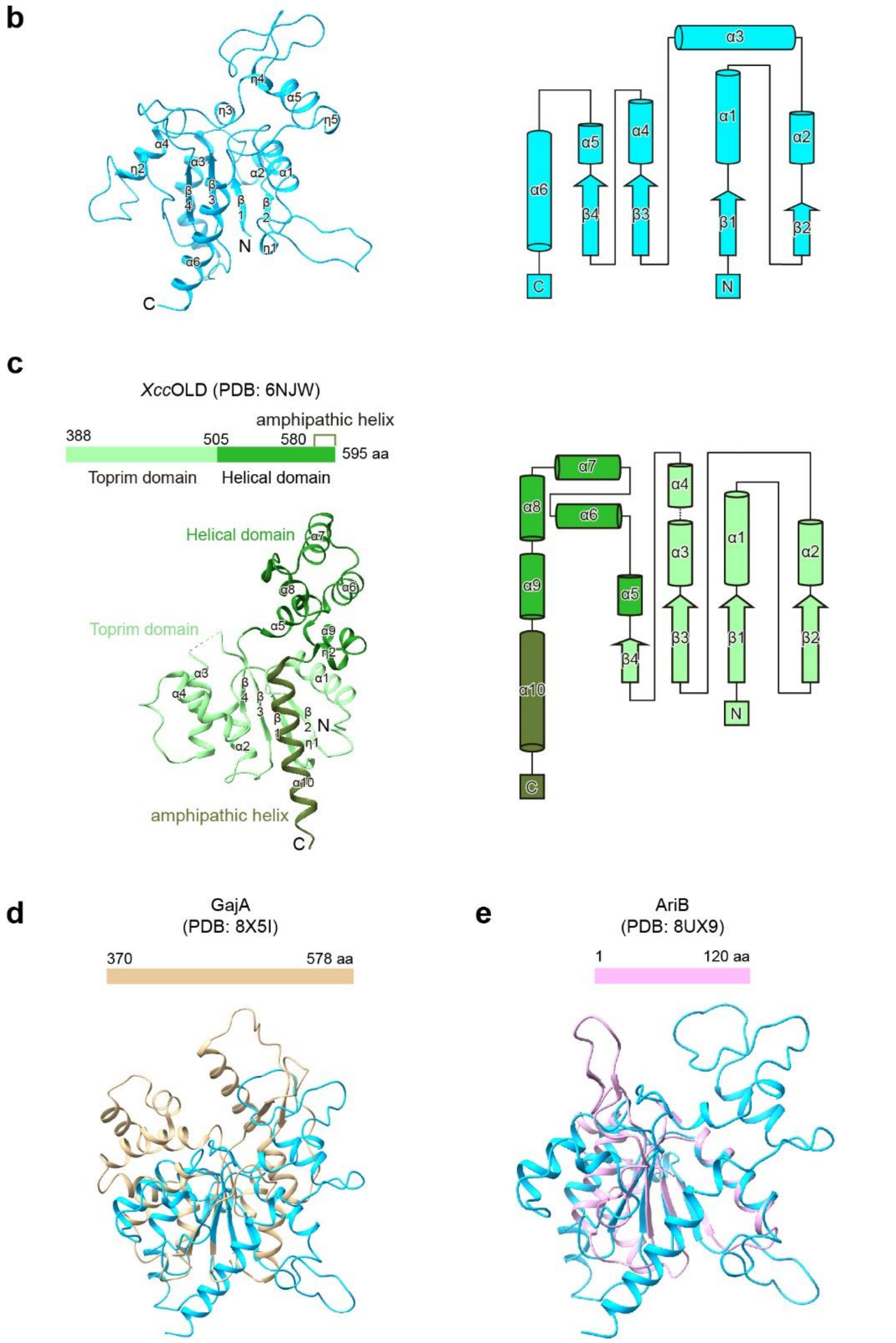

Supplementary information, Fig. S8 Superposition of various Toprim domains. a Comparison table of top-scoring Eco2 Toprim homologs through DALI analysis. **b** Cryo-EM structure and topology diagram of the Eco2 Toprim domain. **c** Cartoon

representation and topology diagram of class 2 overcoming lysogenization defect (OLD) proteins from *Xanthamonas campestris p.v. campestris* (PDB: 6NJW). **d-e** Superposition of the Eco2 Toprim domain with (**d**) endonuclease GajA of Gabija defense system (PDB: 8X5I; RMSD 6.0 Å over 96 residues) and (**e**) AriB of the PARIS immune system (PDB: 8UX9; RMSD 6.5 Å over 80 residues).

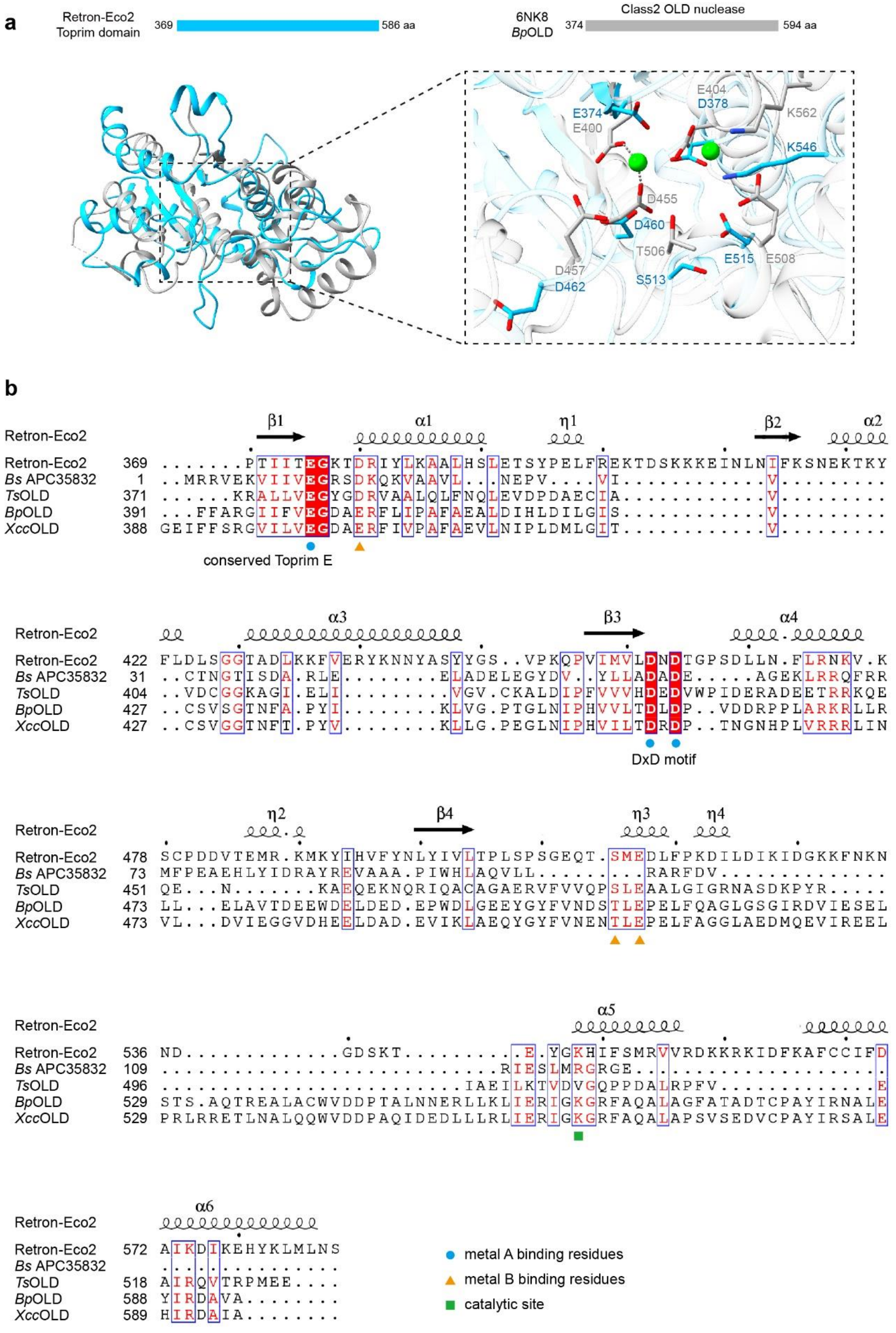

Supplementary information, Fig. S9 Comparison of active-site elements among Toprim domains. a Domain architecture of Eco2 Toprim vs. *Bp*OLD. The top panel provides a schematic representation of the domain architectures of Retron-Eco2 Torim domain and OLD nuclease from *Burkholderia pseudomallei* (PDB: 6NK8). The bottom

panel shows the overlay of active sites from Eco2 Toprim (deep sky blue) and class 2 OLD nuclease from *Burkholderia pseudomallei* (dark gray) (RMSD of 5.39 Å over 112 residues). **b** Alignments of Toprim homologs from various bacterial species, with blue circles and yellow triangles marking metal A and metal B binding sites, and green squares denoting catalytic residues. Sequence alignment was performed using MultAlin.

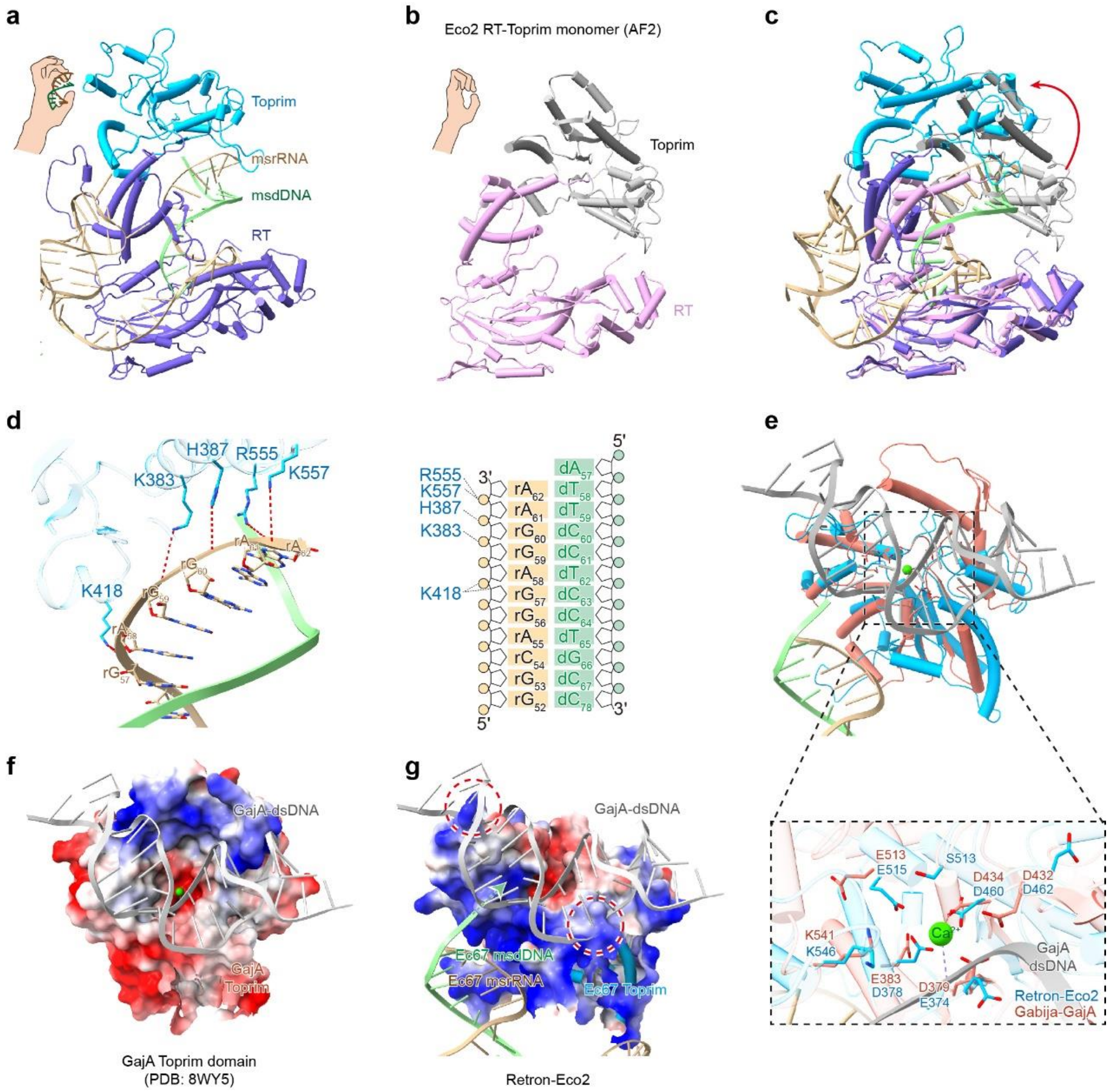

Supplementary information, Fig. S10 Structural analysis of the Retron-Eco2 Toprim domain. a Cryo-EM structure of the RT-Toprim subunits within the Eco2 trimeric assembly. **b-c** AlphaFold2-predicted RT-Toprim (**b**) superimposed on the cryo-EM structure (**c**), showing a "closed" vs. "open" Toprim conformation. **d** Interaction of the Eco2 Toprim with DNA-RNA duplex. Basic residues K383, H387, K418, R555, and K557 in the Toprim domain contact the nucleic-acid backbone. **e** Superposition of the Toprim domain of Retron-Eco2 with the Toprim domain of the Gabija system (PDB: 8WY5; RMSD 6.00 Å over 96 residues). Gabija Toprim domain and dsDNA are shown in salmon and light gray, respectively. **f** Surface representation of the Toprim domain in complex with substrate dsDNA in the Gabija system. **g** Steric hindrance occurs between the potential substrate and the partially resolved DNA part of msDNA within the catalytic pocket of the Eco2 Toprim domain.

Table S1. Cryo-EM data collection, refinement, and validation statistics

	Retron-Eco2<br>(PDB ID, 9LM3)<br>(EMDB ID, EMD-63214)
**Data Collection and Processing**	
Microscope	Krios
Voltage(keV)	300
Camera	Gatan K3
Magnificatin	81,000
Pixel size at detector (Å/pixel)	1.07
Total electron exposure (e–/Å2)	50
Number of frames collected during exposure	32
Defocus range (μm)	-1.2 ~ -2.2
Automation software	EPU v2.9
Energy filter slit width	20eV
Micrographs collected (no.)	5,092
Micrographs used (no.)	4,098
Total extracted particles (no.)	3,108,650
**For each reconstruction:**	
Final particles (no.)	574,158
Point-group	C1
Resolution (global, Å)	
FSC 0.5 (unmasked/masked)	3.5/2.9
FSC 0.143 (unmasked/masked)	3.0/2.6
Resolution range (local, Å)	2.0-4.0
Map sharpening *B* factor (Å2)	112.0
Map sharpening methods	Half-maps correlation
**Model composition**	
Protein	1,746
Ligands/RNA/DNA	225
**Model Refinement**	
Refinement package	PHENIX
- real or reciprocal space	Real space
- resolution cutoff	2.6
Model-Map scores	
- CC	0.83
B factors ($Å_2$)	
Protein residues	58.88
Ligands/RNA/DNA	49.96
R.m.s. deviations from ideal values	
Bond lengths (Å)	0.004
Bond angles (°)	0.561
**Validation**	
MolProbity score	1.74
CaBLAM outliers	5.05
Clashscore	3.47
Poor rotamers (%)	1.47
C-beta deviations	0
**Ramachandran plot**	
Favored (%)	92.33
Outliers (%)	0
